# XSim version 2: simulation of modern breeding programs

**DOI:** 10.1093/g3journal/jkac032

**Published:** 2022-03-04

**Authors:** Chunpeng James Chen, Dorian Garrick, Rohan Fernando, Emre Karaman, Chris Stricker, Michael Keehan, Hao Cheng

**Affiliations:** 1 Department of Animal Science, University of California, Davis, CA 95616, USA; 2 Massey University, Palmerston North 4442, New Zealand; 3 Department of Animal Science, Iowa State University, Ames, IA 50010, USA; 4 Center for Quantitative Genetics and Genomics, Aarhus University, Aarhus 8830, Denmark; 5 agn Genetics GmbH, Davos-Dorf, Graubünden 7260, Switzerland

**Keywords:** simulation, breeding programs, genetic evaluation

## Abstract

Simulation can be an efficient approach to design, evaluate, and optimize breeding programs. In the era of modern agriculture, breeding programs can benefit from a simulator that integrates various sources of big data and accommodates state-of-the-art statistical models. The initial release of XSim, in which stochastic descendants can be efficiently simulated with a drop-down strategy, has mainly been used to validate genomic selection results. In this article, we present XSim Version 2 that is an open-source tool and has been extensively redesigned with additional features to meet the needs in modern breeding programs. It seamlessly incorporates multiple statistical models for genetic evaluations, such as GBLUP, Bayesian alphabets, and neural networks, and it can effortlessly simulate successive generations of descendants based on complex mating schemes by the aid of its modular design. Case studies are presented to demonstrate the flexibility of XSim Version 2 in simulating crossbreeding in animal and plant populations. Modern biotechnology, including double haploids and embryo transfer, can all be simultaneously integrated into the mating plans that drive the simulation. From a computing perspective, XSim Version 2 is implemented in Julia, which is a computer language that retains the readability of scripting languages (e.g. R and Python) without sacrificing much computational speed compared to compiled languages (e.g. C). This makes XSim Version 2 a simulation tool that is relatively easy for both champions and community members to maintain, modify, or extend in order to improve their breeding programs. Functions and operators are overloaded for a better user interface so they may concatenate, subset, summarize, and organize simulated populations at each breeding step. With the strong and foreseeable demands in the community, XSim Version 2 will serve as a modern simulator bridging the gaps between theories and experiments with its flexibility, extensibility, and friendly interface.

## Introduction

Computer simulation is an efficient approach to design, evaluate, and optimize breeding programs. Simulation usually acts as a bridge between theories and experiments to provide development and preliminary validation of theories as well as planning guidelines on empirical experiments. Instead of risking years real world effort, new breeding strategies supported by quantitative genetics theories can mostly be evaluated in a day through simulations with proper assumptions and an appropriate tool.

Integration of various sources of big data including phenotypic, pedigree, genomic, biological, and environmental information for genetic improvement in modern animal and plant agriculture requires an efficient and flexible simulator to both simulate and compare alternative breeding programs using all available information. Such a simulator needs to accommodate state-of-the-art statistical models for genetic evaluation.

As an open-source tool, the initial version of XSim (Cheng *et al.* 2015a), in which a strategy is developed to drop-down positions and origins of chromosomal segments rather than every allele state, is able to efficiently simulate descendants stochastically to sequence level resolution. It has mainly been used to validate genomic selection methods. We have extensively upgraded XSim to incorporate a portfolio of new features to simulate modern breeding programs. In XSim Version 2 (XSimV2), we have seamlessly incorporated “JWAS” (Cheng *et al.* 2018a) an open-source package for genome-enabled analysis to enable multiple statistical models for genetic evaluation, including pedigree-based BLUP (Henderson 1984), GBLUP (Habier *et al.* 2007; VanRaden 2008), the Bayesian Alphabet (Meuwissen *et al.* 2001; Park and Casella 2008; Kizilkaya *et al.* 2010; Habier *et al.* 2011; Erbe *et al.* 2012; Moser *et al.* 2015; Cheng *et al.* 2015b), the multitrait Bayesian Alphabet (Cheng *et al.* 2018b; Gianola and Fernando 2020), single-step methods (Legarra *et al.* 2009; Fernando *et al.* 2014, 2016), as well as Bayesian neural networks (Zhao *et al.* 2021).

Modern breeding programs usually involve complex mating designs, such as crossbreeding in cattle and the use of multiparent populations in plants. XSimV2 is able to effortlessly mimic these designs by allowing the creation of novel mating plans together with modern biotechnology such as using double haploids (DHs) in plants and embryo transfer in animals. In addition, XSimV2 can preload up-to-date information on reference genomes for multiple major breeding species (e.g. cattle, pigs, rice, maize) such that genomic locations and recombination hot or cold spots can be simulated. XSimV2 is implemented in the modern programming language Julia (Perkel 2019), which retains the readability of dynamic languages such as R (R Core Team 2020) and Python (Van Rossum and Drake 1995) but approaches the computing speed of compiled languages such as C (Kernighan and Ritchie 1988). This makes XSimV2 a simulation tool that is relatively easy for both champions and community members to maintain, modify, or extend in order to improve their breeding programs.

In this article, we will illustrate the latest software interface for XSimV2 including the manner in which genomes and phenomes are defined, the approach for generating founders, the methods for choosing the type of mating, the integration of selection based on real-time genetic evaluations, and others. We demonstrate the extensibility with case studies from both animal and plant breeding. The documentation of XSimV2 can be found on the GitHub repository (https://github.com/reworkhow/XSim.jl, last accessed date: Feb. 22nd, 2022).

## Software interface description

In this section, we first describe how the genome and phenome to be simulated are specified. Then, we show how the founders of the population to be simulated can be generated. Following this, we describe how different mating schemes can be specified, including random mating and mating based on phenotypes or genetic evaluations. The descriptions below are followed by Julia code examples (in gray boxes), where the lines beginning with “#” are comments.

### Defining the genome and phenome

A simulation is initialized by defining the genome and phenome with the functions build_genome() and build_phenome(). Multiple parameters in build_genome() can be used to define the genome, including numbers of chromosomes and loci, genetic position, physical position and the minor allele frequency (MAF) of each locus, mutation rates, and genotyping error rates. Phenome-related parameters can be defined in the build_phenome() function including positions and effects of causal variants [quantitative traits loci (QTL)], genetic variance-covariance matrices, residual variance-covariance matrices, phenotypic variance-covariance matrices, or heritability. The genome can also be user defined by providing a map file, in which each row represents one locus. The valid header names include “chr,” “cM,” “bp,” and “maf,” which refer to chromosome identification codes, positions in centimorgans (genetic position), positions in base pairs (physical position), and MAFs, respectively:

# Define a genome with a user-provided file

# An example map.csv file is in the Appendix.

# Header in map.csv:”chr ” , ” cM ” , ” bp ” , and ” maf”

build_genome(”map.csv”)

Note that XSimV2 uses genetic position information to simulate crossover events, and thus, this information encodes recombination hot or cold spots. If information on genetic positions is not provided by the user, XSimV2 can infer genetic positions from physical positions provided using the preloaded genetic and physical representation of reference genomes in XSimV2 through the species argument in build_genome(). XSimV2 preloads genetic and physical maps reported in literature for multiple major livestock and crop species, including cattle (Arias *et al.* 2009), pig (Tortereau *et al.* 2012), rice (Kurata and Yamazaki 2006), and maize (Portwood *et al.* 2019). If neither genetic positions nor studied species are provided, chromosomes are assumed to have a length of 1 Morgan and the relationship between physical and genetic positions are assumed to be linear.

Phenomics is another aspect of the simulation, and it defines how traits are simulated in XSimV2. The allele substitution effects of QTL can be defined by adding columns with eff_ prefixes to the map files used in build_genome(). For example, when there are two traits being simulated, columns eff_1 and eff_2 are added to assign the QTL effects. A zero value should be assigned for a locus that is not QTL. QTL with pleiotropic effects can also be simulated in multitrait simulation. An example of the map file is shown in the Appendix.

# Define a phenome with heritability 0.3 by a user- provided file

# Header in map.csv: ” eff_” prefixed for marker effects

build_phenome(”map.csv”, h2 = 0.3)

Quick Start approaches are available in XSimV2 to allow users to quickly set up their genome and phenome in order to focus on the simulated schemes for proof of concept (more details are in documentation). One example is for a user to define the genome and phenome by providing number of chromosomes, number of loci, numbers of QTL for each trait, genetic variances, and heritability. Genetic positions of all loci will be uniformly distributed along the genome. QTL effects and environmental effects will be sampled from a standard normal distribution. These effects will be scaled and transformed to obtain the predefined genetic variance and heritability. By default, the genetic variance vg and the residual variance ve are assumed to be scalars in single-trait simulation and diagonal matrices in multitrait simulation. Correlations are introduced by assigning nonzero values to the off-diagonal elements of vg and/or ve in multitrait simulation.

The example below shows an approach to simulate two correlated traits controlled by 2 and 1 QTL among 4 SNPs on 2 chromosomes. The traits are simulated to have heritability of 0.3 and 0.8, respectively, with uncorrelated residual effects.

# A quick start of genome with 4 loci on 2 chromosomes

build_genome(n_chr = 2,

       n_loci = 2)

# Phenome with 2 correlated traits of heritability 0.3 and 0.8, controlled by 2 and 1 QTL, respectively.

build_phenome([2, 1],

         vg = [1 .5

          .5 1],

         h2 = [0.3, 0.8])

### Generating founders

In XSimV2, a Julia object, Cohort, was designed to represent a group of individuals and to store their heterogeneous information of simulated genotypes, pedigree, and breeding values. Users can obtain the Cohort object either through the function Founders(), which takes an integer as the input argument specifying the number of simulated founders, or from the results of select or mate functions, which will be described in the later sections. If a cohort is created by Founders() without known haplotypes, the cohort’s haplotypes are simulated by sampling haplotypes of each locus from a Bernoulli distribution with the event probability equals to 1 - MAF. The codes below demonstrate how to initialize a cohort containing ten simulated founders. Note that, as will be shown in the Case Studies section, random mating over a number of generations may be required to generate linkage disequilibrium (LD).

# Generate 10 founders and get a cohort object

cohort_A = Founders(10)

Alternatively, users can specify the genomes of all the founders and directly control their relatedness. This requires users to have known haplotypes or genotypes for founders, which XSimV2 can read from a text file. The haplotypes should have individuals recorded by row with two columns per locus, recording the paternal and maternal alleles at this locus. The haplotype is coded as 0 or 1 to represent the existence of a reference allele. If genotypes are provided, haplotypes will be further inferred from genotypes randomly. In the genotype file, alleles are coded as 0, 1, and 2 to represent the allele dosage, and these values should be arranged as individuals (rows) by loci (columns). Missing haplotypes and genotypes can be denoted as −1 or 9.

# Generate founders from a known haplotype file

# An example haplotypes.csv file is in the Appendix

cohort_B = Founders(”haplotypes.csv”)

### Mating

Many mating schemes conceptually consist of parents that are sampled from two cohorts (cohorts A and B). XSimV2 allows a parent from cohort A to be mated with a specified number of parents from cohort B. The function mate() takes cohort A and B, which both are Cohort objects, as the first two arguments, it can adapt to different scenarios with multiple arguments: nA common parents will be randomly selected from cohort A, and each common parent will mate with nB_per_A individuals randomly sampled from cohort B. Individuals will be sampled with replacement from cohorts A or B depending on if the arguments replace_A or replace_B are set to true. From a pair of parents sampled from two cohorts, n_per_mate offspring will be reproduced with ratio ratio_malefemale of male over female.

For example, in the mating scheme below, we have 5 sires from cohort A, and each sire is mated with 10 dams from cohort B, which are sampled with replacement, to generate 1 progeny with the 1:1 sex ratio.

# A mating function example

args = Dict(:nA => 5,

     :nB_per_A => 10,

     :replace_A => false,

     :replace_B => true,

     :n_per_mate   => 1,

     :ratio_malefemale => 1)

male, female = mate(cohort_A, cohort_B; args…)

The default value for ratio_malefemale is false (i.e. a zero value), and only one Cohort object will be returned if ratio_malefemale is set to false or 0. This mating function is versatile to accommodate most mating schemes (e.g. random mating, diallel crosses, and selfing) by assigning different arguments in mate().

A “random mating” scheme between two cohorts is presented in the example below: Random mating between cohort A and B is performed without replacement, in which 1 progeny is generated from each mating. By default, if only the first two arguments (i.e. cohort A and B) are included in the mate() function, the random mating scheme described above is performed. This scheme can also be expressed by using the overloaded operator “*” as will be shown in the “overloaded operators and functions” subsection.

# Random mating scheme

args = Dict(:nA => cohort_A.n,

:nB_per_A => 1,

:replace_A => false,

:replace_B => false,

:n_per_mate => 1)

offspring = mate(cohort_A, cohort_B; args…)

# Equivalent results without providing any argument

offspring = mate(cohort_A, cohort_B)

# Equivalent results by specifying scheme argument

offspring = mate(cohort_A, cohort_B, scheme =“random”)

# Equivalent results with overloaded operator ’*’

offspring = cohort_A * cohort_B

In the “diallel cross” scheme, each individual from cohort A mates with all individuals from cohort B. In the example below, each individual from cohort A is mated to all individuals in cohort B with one offspring from each mating.

# Diallel cross mating scheme

args = Dict(:nA => cohort_A.n,

:nB_per_A => cohort_B.n,

:replace_A    => false,

:replace_B => false,

:n_per_mate => 1)

offspring = mate(cohort_A, cohort_B; args…)

# Equivalent results by specifying scheme argument

offspring = mate(cohort_A, cohort_B,

scheme = “diallel cross”)

In the “selfing” scheme, self-fertilization (selfing) is performed if the argument scheme =“selfing” in mate(). For example, if one wants to produce 10 families of 50 individuals each from selfing of 10 individuals from cohort A, the mating scheme can be defined as:

# Selfing mating scheme

args = Dict(:nA => 10,

:replace_A => false,

:n_per_mate => 50,

:scheme => “selfing”)

offspring = mate(cohort_A; args…)

As an alternative to specifying the mating scheme, users can provide a pedigree that identifies the parents for each individual. This is done by providing a pedigree file in the mate() function.

# Pedigree mating scheme

# An example pedigree.csv file is in the Appendix

cohort = mate(”pedigree.csv”)

This option in mate() function allows XSimV2 to simulate data from actual pedigrees.

### Selection based on real-time genetic evaluations

XSimV2 allows a cohort of individuals to be selected based on phenotypes (i.e. mass selection) or estimated breeding values (EBV) from real-time genetic evaluations. The selection is implemented by the function select() taking a Cohort object as the first argument, and the number of individuals to be selected from the input cohort as the second argument. By default, mass selection is conducted and the argument criteria = “phenotypes” is used. To estimate breeding values, a genome-enabled analysis package “JWAS” (Cheng *et al.* 2018a) was incorporated into XSimV2. Multiple methods are available for genetic evaluations, including pedigree-based BLUP (Henderson 1984), GBLUP (Habier *et al.* 2007; VanRaden 2008), Bayesian Alphabet (Meuwissen *et al.* 2001; Park and Casella 2008; Kizilkaya *et al.* 2010; Habier *et al.* 2011; Erbe *et al.* 2012; Moser *et al.* 2015; Cheng *et al.* 2018b; Gianola and Fernando 2020), and single-step methods (Legarra *et al.* 2009; Fernando *et al.* 2014) for single-trait and multiple-trait analyses (Cheng *et al.* 2018b; Gianola and Fernando 2020), as well as Bayesian neural networks (Zhao *et al.* 2021).

After EBV (or phenotypes in phenotypic selection) are obtained, a selection index can be used to combine information on multiple traits for selection. The argument weights can be assigned as weights for traits in the selection index. Note that positive or negative weights enable selection in either ascending or descending order of EBV.

In the example below, two correlated traits are simulated to be controlled by 10 and 20 QTL out of 50 locus on one chromosome, respectively. Individuals are selected based on EBV. Multitrait GBLUP is used to obtain EBV for both traits by default. The selection index weights for these two traits are set to 3.0 and −2.0. If no weight are assigned, weights are assumed to be 1 for each trait.

# Genetic evaluation

args = Dict(:criteria   => “EBV ”,

:methods => “GBLUP ”,

:weights => [3.0, -2.0])

offspring = select(cohort_A, 50; args…)

### Breed

Here, we introduce a function breed() as a wrapper function that combines the functions for mate() and select(). The value of n_gens defines how many generations are simulated, and n_select_A and n_select_B define how many descendants are selected to form the cohort A and cohort B in the next generation, respectively. For example, we can have 10 sires (cohort A) and mate each sire with 5 dams (cohort B) for 3 generations. And in each generation we mass select 10 male offspring as sires and all female offspring as parents for the next generation. The code for such a breeding scheme is given below:

# Mating and selection cross 5 generations

args = Dict(# mating arguments

:nA => 10,

:nB_per_A => 5,

:replace_A => false,

:replace_B => true,

:n_per_mate => 1,

:ratio_malefemale => 1.0,

# selection arguments

:criteria => “EBV ”,

:methods => “GBLUP ”,

# breeding arguments

:n_gens => 3,

:n_select_A => 10)

# Breed cohorts based on the defined arguments

cohort_A, cohort_B = breed(cohort_A, cohort_B; args…)

# The result is equivalent to the following mate-select iterations:

for _ in 1:3

males, females = mate(cohort_A, cohort_B; args…)

cohort_A = select(males, sires.n; args…)

cohort_B = females

end

### Species-specific features

Several species-specific features are included in XSimV2. Two examples are shown in this section including DHs in plant breeding and embryo transfer in animal breeding.

DHs production is an important tool in plant breeding for reducing costs and speeding up the fixation of inbred lines. To generate breeding lines with high homozygosity, plant breeders may use a number of generations of selfing, back-crossing, or advancing generations by single seed descent (SSD). Instead, the production of DH breeding lines can derive 100% homozygous individuals in just one generation. In XSimV2, users can call the function get_DH() and generate DH lines as below:

# The offsprings “ DHs” have the same population size as

the parents “ cohort”

DHs = get_DH(cohort)

Ovum pick-up is a technology, where a cow is super ovulated and then the unfertilized eggs are flushed out and in vitro fertilized with potentially different sires. It can be straightforward to implement these technologies in XSimV2 by defining cows (dams) as cohort A, which will be treated as a common parent. The mating scheme can be setup as:

# Example to demonstrate ovum pick-up in cattle breeding

args = Dict(:nA => 5, # 5 dams in total

:nB_per_A => 10, # mate each dam with 10 sires

:replace_A => false,

:replace_B => true,

:n_per_mate => 1)

offspring = mate(dams, sires; args…)

### Overloaded operators and functions

In XSimV2, as cohorts are the basic unit in both of the functions mate() and select(), we made a Cohort “object” for a collection of individuals and overloaded multiple base operators and functions in XSimV2. For example, users can simply use subset or + operators to get a subset of a cohort or to concatenate multiple cohorts into one larger group. In the example below, we concatenate the first 5 individuals in cohort A, and all individuals in cohort B into one single large cohort C.

# Concatenate multiple cohorts

cohort_C = cohort_A[1:5] + cohort_B

The base functions sort() and sample() are also overloaded for Cohort. Users can sample random individuals from a cohort, or sort individuals using similar syntax (more examples are available in the documentation).

# Sample 5 individuals from a cohort without replacement

new_cohort = sample(cohort_A, 5, replace = false)

# Sort the cohort by true breeding values (BV). “ BV” is the default value.

sort_cohort = sort(cohort_A, by = ”BV”)

# Or sort the cohort based on their pedigree in an order from the oldest to the youngest. Other options include estimated breeding values (EBV) or phenotypes ( *e.g.* y1).

sort_cohort = sort(cohort_A, by = ”pedigree”)

## Case studies

### Rotational cross-breeding in cattle

**Fig. 1.**
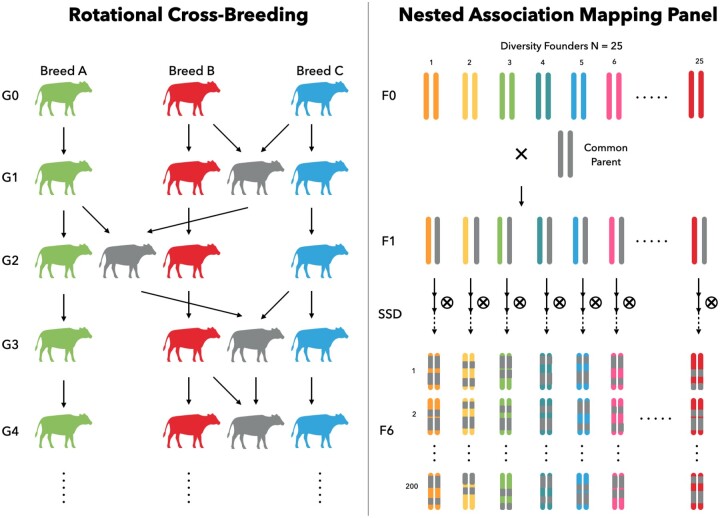
Case studies of mating schemes: rotational cross-breeding in animal breeding (left), and NAM panel in plant breeding (right).

An example of rotational cross-breeding is shown in Fig. 1. First, a cattle founder population is initialized, either based on real haplotypes or genotypes (described in the section *Generating Founders*), or simulated based on allele frequencies, assuming linkage and Hardy–Weinberg equilibria, with random mating over a number of generations to generate LD. For example, we might start with a population of 1,500 individuals. We let them randomly mate for 1,000 discrete (nonoverlapping) generations. In order to expand the LD range, we subject the population to a bottleneck that reduces its size to 100 individuals, and then allow random matings for another 15 generations. This approach is similar to that proposed in Habier *et al.* (2013), which was used to simulate a similar LD pattern to that observed in real dairy cattle (de Roos *et al.* 2008). By default, the mate() function can have all individuals from the input cohort mating randomly with each other:

# Build Genome and Phenome

build_genome(”map.csv , ” species = “cattle”)

build_phenome(”map.csv , ”

vg = [1 .5; .5 1],

h2 = [0.3, 0.7])

# Initialize a population with 1,500 parents of linkage and Hardy Weinberg equilibria

parents = Founders(1500)

# Let parents random mate with each other for 1,000 generations

for _ in 1:1000

parents = mate(parents)

end

# Drop the population size to 100 individuals and continue the random mating for another 15 generations

for _ in 1:15

parents = mate(parents[1:100])

end

sires_base = dams_base = parents

Second, the ancestors of what will become three pure breeds (breeds A, B, and C) are generated in generation $G_{0}$ from the parental population through random mating for 10 generations. In breed A, 50 sires and 500 dams are randomly selected, and each sire is mated with 10 dams. The ratio of male to female offspring is 1. In both breeds B and C, 100 sires and 2000 dams are randomly selected, and each sire is mated with 20 dams. Thus, three pure breeds are simulated, where breed A represents a small population of 50 males and 500 females, whereas both breeds B and C have 100 males and 2000 females.

# Simulate three pure breeds

args_A = Dict(# Mating

:nA => 50,

:nB_per_A => 10,

:n_per_mate => 2,

:ratio_malefemale => 1,

# Selection

:criteria => “random ”,

# Breeding

:n_gens => 10,

:n_select_A => 50,

:n_select_B => 500)

args_BC = Dict(# Mating

:nA => 100,

:nB_per_A => 20,

:n_per_mate => 2,

:ratio_malefemale => 1,

# Selection

:criteria =>“random ”,

# Breeding

:n_gens => 10,

:n_select_A => 100,

:n_select_B => 2000)

# Breed A, B, and C

sires_A, dams_A = breed(sires_base, dams_base; args_A…)

sires_B, dams_B = breed(sires_base, dams_base; args_BC…)

sires_C, dams_C = breed(sires_base, dams_base; args_BC…)

Third, we create first-cross-offspring, which contain 2,000 individuals, using all 100 sires from breed B and 1,000 dams from breed C as two mating cohorts. Each sire will mate with 10 dams and produce two offspring which leads to 1,000 male and 1,000 female offspring at generation $G_{1}$. These first-cross-animals will be sires and dams in the next generation $G_{2}$. We also continue pure-bred matings to retain subsequent generations of each of the three breeds. In $G_{2}$, we use all 50 sires from breed A and cross each of them with 20 dams from $G_{1}$. And we can generate another 1,000 males and 1,000 females as parents for the next round. In $G_{3}$, all 100 sires from breed C will mate with dams from $G_{2}$ and produce another 2,000 offspring. The sire for the next generation will be back to breed B and the rotational crossbreeding cycle continues. The described process can be adequately expressed in XSimV2:

# Rotation parameters

args_XA = Dict (:nA => 50,

:nB_per_A => 20,

:n_per_mate => 2,

:ratio_malefemale => 1)

args_XBC = Dict(:nA => 100,

:nB_per_A => 10,

:n_per_mate => 2,

:ratio_malefemale => 1)

args_A[:n_gens] = 1

args_BC[:n_gens] = 1

# Rotation (G1)

sires_A1, dams_A1  = breed(sires_A, dams_A; args_A…)

sires_B1, dams_B1 = breed(sires_B, dams_B; args_BC…)

sires_C1, dams_C1  = breed(sires_C, dams_C; args_BC…)

males_G1, females_G1 = mate(sires_B, dams_C;

args_XBC…)

# Rotation (G2)

sires_A2, dams_A2 = breed(sires_A1, dams_A1; args_A…)

sires_B2, dams_B2 = breed(sires_B1, dams_B1; args_BC…)

sires_C2, dams_C2 = breed(sires_C1, dams_C1; args_BC…)

males_G2, females_G2 = mate(sires_A1, females_G1;

args_XA…)

# Rotation (G3)

sires_A3, dams_A3 = breed(sires_A2, dams_A2; args_A…)

sires_B3, dams_B3 = breed(sires_B2, dams_B2; args_BC…)

sires_C3, dams_C3 = breed(sires_C2, dams_C2; args_BC…)

males_G3, females_G3 = mate(sires_C2, females_G2;

args_XBC…)

### Nested association mapping from inbred plants

An example of nested association mapping is shown in Fig. 1. The NAM design (Buckler *et al.* 2009) is useful in plant breeding for its statistical power in detecting QTLs (Scott *et al.* 2020). The demonstrated genotype below was collected from a real maize association panel (Wang and Zhang 2020) and was preloaded in XSimV2. Founders of the NAM panel are determined by 25 diversity founders and 1 common parent sampled from this preloaded dataset.

# Load demo data

data_map = DATA(”maize_map”)

data_snp = DATA(”maize_snp”)

# Build genome using the real data

build_genome(data_map)

# Simulate a trait controlled by 30 QTLs

build_phenome(30)

# Randomly sample 26 founders to become the base population

founders = Cohort(data_snp,

n = 26, random=true)

common_parents = founders[1]

diverse_parents = founders[2:26]

We let each founder mate with the common parent separately to generate 25 families in the generation $F_{1}$. Later, each family is advanced by SSD to the 6th generation and derive 200 recombinant inbred lines (RILs). Overall, we can have a NAM population with 25 families containing 200 RILs each through the following code:

# Create an empty cohort to be concatenated with newly generated offspring

F1 = Founders()

# Cross each diverse parent with the common parent

for parent in diverse_parents

F1 += common_parents * parent

end

# Each family produce 200 RILs to derive NAM population

args = Dict(# Mating

:n_per_mate => 10,

:scheme =>“selfing ”,

# Selection

:criteria =>“phenotypes ”,

# Breed

:n_gens => 4,

:n_select => 1)

NAM = Founders()

for family in F1

F2 = mate(family, n_per_mate = 200,  scheme=”selfing”)

for seed in F2

# single seed decent

NAM += breed(seed; args…)

end

end

## Discussion

An efficient way to design, evaluate, and optimize modern breeding programs with complicated mating systems and state-of-art biotechnologies and statistical models is provided by XSimV2. Beyond the case studies presented in this article, XSimV2 can be flexibly extended to desired settings with the aid of its modular construction. Core functions of mate and select cover the two major breeding steps and can function as independent modules. It is possible to reassemble them in arbitrary combinations and achieve as many possible designs as each user desires. XSimV2 is extensible to accommodate newly released technologies and statistical models.

Breeding programs of diploid species can be adequately simulated in XSimV2. When the species is polyploid, users can add an alphabet to chromosome codes (e.g. 1A, 1B, 2A, 2B,…, for allotetraploid) in the map file to indicate their subgenome for allopolyploid species. XSimV2 will treat chromosomes from different subgenomes as independent pairs of chromosomes. Otherwise, users can always extend it to other polyploid behaviors with extensible development in XSimV2.

The initial version of XSim (Cheng *et al.* 2015a), an open-source software tool, introduced an efficient approach to simulate Mendelian inheritance. In this approach, a chromosome of a nonfounder was represented by a list of the starting positions and origins of founder haplotypes. Meiotic crossovers between a pair of maternal and paternal chromosomes gives rise to a new list of starting positions and origins, which represents a new chromosome (Cheng *et al.* 2015a). A chromosome of one Morgan on average has one crossover during meiosis, and thus, in the first generation of nonfounders, the position-origin list, representing such a chromosome, would on average have a length on only one, regardless of the number of loci being simulated on the chromosome. When a large number of loci are simulated, this approach to simulation results in a tremendous saving in storage space relative to recording the allele state at each locus that is simulated on the chromosome. Given the allele states of the founder haplotypes and the position-origin list of a nonfounder chromosome in any generation, the allele states of the nonfounder chromosome can be efficiently generated without dropping down allele states each generation. Thus, when allele states are not needed in each generation, this approach to simulation results in tremendous savings in storage and computing time.

For the current setting, tens of thousands of individuals in each population with thousands of markers recorded can be simulated in few minutes per generation on a personal laptop (MacBook 16″ with 2.6 GHz 6-Core Intel Core i7 and 16GB 2667 MHz DDR4). However, the computing speed and memory efficiency of XSimV2 still has room for further improvement. For example, when we simulate a population, computations for each individual at the same generation are mostly independent. Additional computing resources, if applicable, can be utilized to resolve the intensive simulation with the aid of parallel computing. Large matrices, such as genotypes, can be stored in a sparse matrix coded in 8-bit integers instead of a regular dense matrix to improve memory efficiency.

Several breeding program simulators have been developed, e.g. QMSim (Sargolzaei and Schenkel 2009), SBVB (Pérez-Enciso *et al.* 2017), MoBPS (Pook *et al.* 2020), and AlphaSim (Faux *et al.* 2016; Gaynor *et al.* 2021). These simulators are capable of fulfilling needs in practical scenes with their strengths, such as efficient algorithms in simulating Mendelian inheritance (Pérez-Enciso *et al.* 2017), open-source environments for better extensibility (Faux *et al.* 2016; Pook *et al.* 2020), versatile models in genetic evaluation (Pérez-Enciso *et al.* 2017), or high flexibility in mating design (Faux *et al.* 2016; Pook *et al.* 2020). However, current community is still in strong need for all the mentioned merits in a single software platform to design and simulate modern breeding problems. In conclusion, with the foreseeable demands in the community, XSimV2 can serve as a modern simulator to bridge those features missing in alternative software, and within the context of a friendly environment, with strong flexibility, and readily extended as we presented in this article.

## Data availability

The authors state that all data necessary for confirming the conclusions presented in the article are represented fully within the article. The documentation of XSimV2 can be found on the GitHub repository (https://github.com/reworkhow/XSim.jl).

## Funding

This work was partially supported by the United States Department of Agriculture, Agriculture and Food Research Initiative National Institute of Food and Agriculture Competitive Grant No. 2018-67015-27957 and No. 2021-67015-33412.

## Conflicts of interest

None declared.

## Appendix

### Data formats

In the “map.csv” file used in build_genome() and build_phenome(), each row represents one locus. The valid header names, which can be read by build_genome(), include “chr,”” cM,” “bp,” and “maf,” which refer to chromosome codes, positions in centimorgans (genetic position), positions in base pairs (physical position), and minor allele frequencies (MAFs). The valid header names, which can be read by build_phenome() is column names with eff_ prefixes to specify QTL effects of multiple traits. In the example below, we have 4 markers on 2 chromosomes with specific MAFs, physical, and genetic positions. The first marker are simulated as a pleiotropic QTL with nonzero effects, and the remaining markers are not treated as QTL, for which zero effects are assigned.

To define genome and phenome by files, the files should be formatted as rows of observations with valid header names. Assuming we want to simulate a genome with four markers located on two chromosomes, and there are two traits of interest with known QTL effects. We can put chromosome codes, physical positions, and genetic positions in the column chr, bp, cM, respectively. And use columns with eff_ prefixes to specify QTL effects of two traits. The file for the described scenario is formatted as:

# map.csv

id, chr, bp, cM, MAF, eff_1, eff_2

snp_1,1,1818249,50.8,0.5,1.5,2.8

snp_2,1,6557697,80.3,0.5,0.0,0.0

snp_3,2,2298800,39.2,0.5,0.0,0.0

snp_4,2,5015698,66.3,0.5,0.0,0.0

To initialize founders using known haplotypes, genotypes, or pedigree, users can provide such information in a text file. The haplotypes should have individuals recorded by rows, and have every two columns recording paternal and maternal alleles for each locus. The haplotype is coded as 0 or 1 to represent the existence of a reference allele. If genotypes are provided, haplotypes will be further inferred from genotypes randomly. In the genotype file, alleles are coded as 0, 1, and 2 to represent the allele dosage, and these values should be arranged as individuals (rows) by loci (columns). Missing haplotypes and genotypes can be denoted as −1 or 9. An example for 5 individuals and 4 loci is shown below. If the provided file indicate the pedigree, it should be a three-column file in an order of individual ID, sire ID, and dam ID. ID and pedigree information can be obtained from calling get_pedigree(cohort). The demonstrate example showing 5 individuals with the first 3 are the founders and the rest 2 are reproduced from the first and second individuals.

# haplotypes.csv

1,1,0,0,0,0,1,0

0,0,0,0,1,0,0,0

0,0,0,1,0,0,1,1

1,0,1,0,0,0,1,1

1,1,0,0,1,1,0,0

# genotypes.csv

2,0,0,1

0,0,1,0

0,1,0,2

1,1,0,2

2,0,2,0

# pedigree.csv

1,0,0

2,0,0

3,0,0

4,1,2

5,1,2
